# Impact of Perioperative Lidocaine on Neutrophil Extracellular Trapping and Serum Cytokines in Robot-Assisted Radical Prostatectomy: Randomized Controlled Study

**DOI:** 10.3390/medicina60091452

**Published:** 2024-09-05

**Authors:** Dongho Shin, Jiheon Kim, Subin Lee, Min Suk Chae

**Affiliations:** 1 Department of Urology, College of Medicine, The Catholic University of Korea, Seoul 06591, Republic of Korea; eds8813@naver.com; 2 Department of Anesthesiology and Pain Medicine, Yeouido St. Mary’s Hospital, College of Medicine, The Catholic University of Korea, Seoul 06591, Republic of Korea; mysayjihun@gmail.com (J.K.); 1004shelly@naver.com (S.L.); 3 Department of Anesthesiology and Pain Medicine, Seoul St. Mary’s Hospital, College of Medicine, The Catholic University of Korea, 222, Banpo-daero, Seocho-gu, Seoul 06591, Republic of Korea

**Keywords:** prostate cancer, lidocaine, NETosis

## Abstract

*Background and Objective:* This randomized controlled trial investigated the influence of perioperative lidocaine administration on the postoperative inflammatory response in patients undergoing robot-assisted radical prostatectomy, with the results having potential implications for postoperative recovery and cancer recurrence via neutrophil extracellular trapping (NETosis). *Materials and Methods:* In total, 58 patients with localized prostate cancer were randomly assigned to receive an intravenous infusion of 2% lidocaine or a saline placebo intraoperatively. Serum levels of interleukin (IL)-6, IL-10, and IL-17, tumor necrosis factor(TNF)-α, interferon(IFN)-γ, neutrophil elastase (NE), citrullinated histone3 (CitH3), and myeloperoxidase (MPO) were determined preoperatively and at 24 h postoperatively. Biochemical recurrence (BCR) was assessed over a follow-up period of 2 years. *Results:* The lidocaine group showed a significant change in MPO, a greater reduction in IL-10 level, and a smaller increase in the NE level compared to the placebo group, suggesting a modulatory effect of lidocaine on certain anti-inflammatory and neuroendocrine pathways. No significant difference in the BCR rate was observed between the two groups. *Conclusions:* Perioperative lidocaine administration selectively modulates certain inflammatory and neuroendocrine responses after robot-assisted radical prostatectomy surgery, potentially influencing recovery outcomes. These findings highlight the need for further investigations of the role of lidocaine in Enhanced Recovery After Surgery protocols, particularly in oncologic surgeries.

## 1. Introduction

Prostate cancer is one of the most common malignancies among men worldwide, with its incidence varying across geographic regions and populations [1]. The management of localized prostate cancer is individualized according to the disease characteristics, health status, and treatment preferences of patients. The management options include active surveillance for low-risk cases, which involves close monitoring without immediate intervention, as well as aggressive treatments such as radical prostatectomy, radiation therapy, and hormone therapy for high-risk or advanced diseases [2].

Lidocaine, a safe and effective anesthetic agent, is widely utilized for anesthesia and pain management. It has diverse routes of administration, including mucosal and skin application, intramuscular injection, and intravenous injection [3]. Furthermore, it has also demonstrated remarkable efficacy in a distinct clinical domain, cardiology, particularly for the control of arrhythmias. These applications of lidocaine contribute to the early recovery of postoperative patients, and the drug has been integrated into Enhanced Recovery After Surgery (ERAS) programs at leading medical institutions worldwide [4]. In the field of oncology, intravenous administration of lidocaine during surgery reduces the exacerbation of inflammation and inhibits the growth and progression of cancer [5]. Moreover, ongoing research is investigating the efficacy of lidocaine administration for various types and stages of cancer. 

Recent advancements have highlighted the role of the immune system in cancer progression, with a particular focus on the function of neutrophil extracellular traps (NETs) and their impact on cancer biology. NETosis, the mechanism underlying NET formation, is conventionally implicated in the defense against pathogens and is increasingly being found to be involved in various non-infectious diseases, including cancer [6].

In this study, we explored the effects of inflammatory responses on cancer recurrence in patients undergoing advanced cancer resection surgeries using robot-assisted radical prostatectomy.

## 2. Materials and Methods

### 2.1. Ethical Considerations

The study protocol of this prospective, randomized controlled trial was approved by the Ethics Committee of Seoul St. Mary’s Hospital (KC21MISI0105) on 6 April 2021. The protocol was registered with the Clinical Research Information Service, Republic of Korea (KCT0006084) on 13 April 2021. The study was conducted in accordance with the Declaration of Helsinki. All participants provided written informed consent before enrollment in this study. The patients were enrolled between 13 September 2021 and 20 July 2022. 

### 2.2. Study Participants

The study included adult patients with low or intermediate localized prostate cancer (cT1a–cT2b; Gleason score 2–7; prostate-specific antigen [PSA] ≤ 20 ng/mL), life expectancy > 10 years, and American Society of Anesthesiologists physical status I or II who underwent elective robot-assisted laparoscopic radical prostatectomy. We excluded vulnerable patients who were unable to make decisions, including adolescents aged <19 years and older patients aged > 75 years. Furthermore, we excluded patients with a history of side effects associated with lidocaine use; known cardiac disease characterized by arrhythmias, hypotension (mean blood pressure < 60 mmHg), or bradycardia (<40 bpm/min); bleeding requiring transfusion (i.e., hemoglobin < 7 g/dL); vascular diseases; or pulmonary diseases such as asthma or chronic obstructive pulmonary diseases.

**Figure 1:**
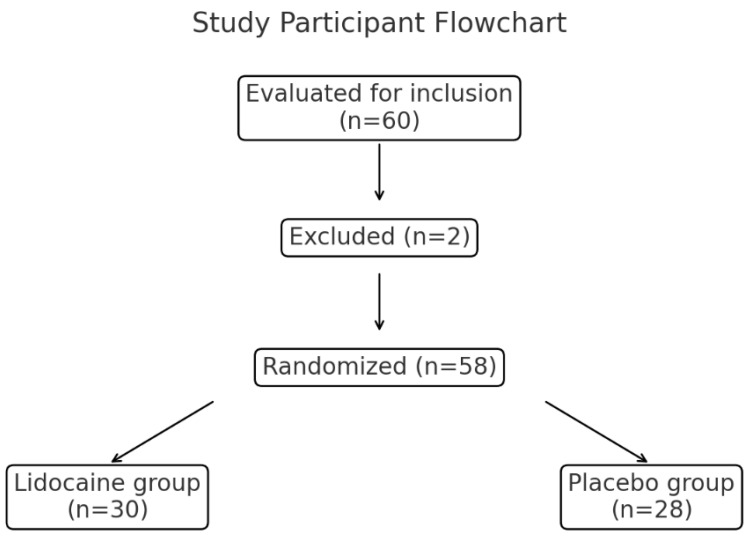
The CONSORT diagram of the study.

In total, 60 patients were evaluated for inclusion in the study, of whom two were excluded due to intraoperative bleeding requiring transfusion. The remaining 58 patients were randomized into the lidocaine group (n = 30) or placebo group (n = 28) (Figure 1).

### 2.3. Randomization and Blinding

Patients were randomized to the lidocaine or placebo group using a web-based random number generator, which allowed for stratified block randomization (www.random.org, accessed on 13 September 2021). A research nurse who was not involved in patient treatment randomized the patients. Sequentially numbered opaque envelopes were opened by medical staff to determine patients’ group assignments. Both patients and surgeons were blinded to the group assignments. Similarly, medical staff involved in postoperative care and outcome evaluation in the post-anesthesia care unit (PACU) and ward were blinded to the group assignments. Drugs were prepared by an anesthesia nurse who was not involved in the outcome assessment. The lidocaine and normal saline used as placebo were rendered indistinguishable and delivered to the operating room. To facilitate objective assessments, anesthesiologists and healthcare providers assessing postoperative outcomes were unaware of the group assignments.

### 2.4. Interventions

The test group received intravenous administration via a central venous line of 2% lidocaine HCL diluted with 20 mL of saline to achieve a volume of 40 mL, resulting in a concentration of 1%. The intervention involved the administration of a 1.5 mg/kg loading dose 10 min after anesthetic induction (phase I), 2 mg/kg/h during surgery (phase II), and 1 mg/kg/h during the first 24 h postoperatively (phase III). To avoid lidocaine-related complications, the dosage was set at ≤300 mg/h, and the rate was set at ≤240 mg/h. As a placebo, the control group received 40 mL intravenous saline via a central venous line, with an administration dose and rate similar to the lidocaine group. 

### 2.5. Surgery and General Anesthesia 

Robot-assisted laparoscopic radical prostatectomy was performed by expert urologists using a robotic-assisted laparoscopic device (Da Vinci Xi System; Intuitive Surgical, Sunnyvale, CA, USA). Patients were positioned in the lithotomy position, and the operative field was disinfected and draped. CO_2_ gas was insufflated into the abdominal cavity to produce pneumoperitoneum with a pressure of up to 15 mmHg via a 12-mm camera trocar inserted through a periumbilical incision. Subsequently, the remaining five trocars, including three 8-mm robotic trocars and 15-mm and 5-mm assisting trocars, were inserted. The intra-abdominal pressure was reduced to 12 mmHg, and the patient was placed in the steep Trendelenburg position at the maximal angle (45°) of the surgical table (Maquet; Rastatt, Baden-Württemberg, Germany). This position was routinely adopted to optimize the surgical view. Intra-abdominal pressure was maintained at 12–15 mmHg during the surgery. At the time of peritoneal closure, the patient was returned to the supine position, and the CO_2_ gas was removed.

Balanced anesthesia was administered alongside standard vital sign monitoring, including electrocardiography, systolic and diastolic blood pressure, heart rate, oxygen saturation, body temperature, and capnography, conducted by attending expert anesthesiologists who were aware of the group allocations but were not involved in subsequent patient care or data collection beyond completing medical records. Anesthesia induction involved the infusion of propofol (1–2 mg/kg; Fresenius Kabi, Bad Homburg, Germany) and 0.6 mg/kg rocuronium (Merck Sharp & Dohme Corp., Kenilworth, NJ, USA), whereas anesthesia maintenance was achieved with 4.0–6.0% desflurane (Baxter, Deerfield, IL, USA) in medical air/oxygen to sustain anesthesia within a bispectral index range of 40–60, ensuring adequate hypnotic depth. Remifentanil (Hanlim Pharm. Co., Ltd., Seoul, Republic of Korea) was continuously infused at a rate of 0.1–0.5 μg/kg/min as appropriate. Rocuronium was administered repeatedly under train-of-four monitoring (more than one twitch). Mechanical ventilator mode adjustments were made to maintain end-tidal CO_2_ between 30 and 40 mmHg. Hypotensive events, defined as systolic blood pressure < 90 mmHg or diastolic blood pressure < 60 mmHg sustained over 5 min, were managed with rescue intravenous ephedrine administration (Daewon Pharm. Co., Ltd., Seoul, Republic of Korea) and/or fluid resuscitation therapy at the discretion of the attending anesthesiologists.

Attending physicians and nurses in the PACU were not involved unless specific surgical complications emerged, such as massive hemorrhage necessitating blood product transfusion, persistent hemodynamic instability such as hypotension requiring continuous vasopressor infusion (e.g., epinephrine or norepinephrine), or fluid resuscitation therapy during or after surgery. All patients were transferred to the ward within 1 h of surgery.

### 2.6. Measurement of Serum Inflammatory Markers 

Serum levels of five cytokines (interleukin [IL]-6, IL-10, IL-17, interferon [IFN]-γ, and tumor necrosis factor [TNF]-α) and three neutrophil extracellular trap (NETosis) markers (myeloperoxidase [MPO], citrullinated histone H3 [CitH3], and neutrophil elastase [NE]) were assessed in patients immediately before induction of general anesthesia (in the operating room) and at 1 day postoperatively (in the ward). Blood samples were collected into test tubes (BD Vacutainer, K2 EDTA; Becton, Dickinson, and Co., Franklin Lakes, NJ, USA) via a central venous line using a sterile technique. Blood samples were transported to the laboratory in an ice-filled container, centrifuged (1500 rpm for 10 min at 4 °C), and frozen at −70 °C until analysis. Serum cytokine levels were determined using sandwich enzyme-linked immunosorbent (ELISA) assays and a human 25-plex antibody bead kit (Invitrogen, Carlsbad, CA, USA). Data were analyzed using the Luminex detection system (200TM; Luminex Corp., Austin, TX, USA). The three NETosis markers were measured utilizing commercially available ELISA kits for MPO (Human MPO; MyBioSource, San Diego, CA, USA; assay range: 0–100 ng/mL), H3Cit (Human H3Cit; MyBioSource; assay range: 0–500 ng/mL), and NE (Human NE; MyBioSource; assay range: 0–128 ng/mL) following the manufacturer’s instructions.

### 2.7. Biochemical Recurrence 

Biochemical recurrence (BCR) was defined as a PSA level ≥ 0.2 ng/mL following radical prostatectomy at the end of the study (at 24 months) [7].

### 2.8. Outcome Assessment

Preoperative and 24-h postoperative blood levels of IL-6, IL-10, IL-17, TNF-α, IFN-γ, NE, CitH3, and MPO were compared. Furthermore, the presence of BCR was assessed. 

### 2.9. Statistical Analyses

To calculate the required sample size, we reviewed the medical records of patients with available IL-6 levels, revealing mean values of 12 and 18 pg/mL in groups that did and did not receive lidocaine during surgery, respectively. The standard deviation for both groups was 8 pg/mL. Assuming a 1:1 allocation ratio to the control and experimental groups, a significance level of 5%, a power of 80%, and a drop-out rate of 10%, the required sample size was 30 participants per group (total of 60 participants).

For categorical variables, frequencies and proportions were determined using the Chi-squared test. For continuous variables, median and interquartile range values were compared using the Mann-Whitney U test. Wilcoxon test and univariate logistic regression analysis were conducted to assess the ability of changes in inflammatory cytokines associated with lidocaine administration to predict BCR. *p*-values < 0.05 were considered indicative of statistical significance. All statistical analyses were carried out using R software (version 4.3.1; R Development Core Team, Vienna, Austria).

## 3. Results 

### 3.1. Baseline Characteristics

From September 2021 to July 2022, 60 patients who underwent robot-assisted radical prostatectomy at Seoul St. Mary’s Hospital were enrolled, of whom two subsequently withdrew from the study. Thus, 58 patients with prostate cancer successfully completed the study.

Table 1 presents a comparison of patient characteristics between the lidocaine (n = 30) and placebo (n = 28) groups. The mean age was similar between the lidocaine and placebo groups (66.43 ± 3.71 and 66.28 ± 5.93 years, respectively; *p* = 0.709). There were no significant differences between the lidocaine and placebo groups in terms of the body mass index (23.21 ± 1.29 and 23.73 ± 4.13 kg/m^2^, respectively; *p* = 0.911), PSA level (6.9 ± 3.3 and 6.7 ± 4.5 ng/mL, respectively; *p* = 0.836), or TNM stage (*p* = 0.556–0.828 for the T2, Nx, N0, and M0 stages). Positive surgical margins were observed in two and one patients in the lidocaine and placebo groups, respectively (*p* = 0.804), suggestive of minimal differences between the groups. BCR occurred in four and three patients in the lidocaine and placebo groups, respectively (*p* = 0.257). There was no significant difference in the time to BCR between the lidocaine and placebo groups (14.5 ± 4.5 and 24 ± 1.0 months, respectively; *p* = 0.754). Furthermore, no statistically significant differences were observed in demographic or clinical characteristics between the lidocaine and placebo groups [8]. Additionally, there were no adverse events such as arrhythmia [9], polycythemia vera, thrombocythemia [10], or seizures in the lidocaine and placebo groups. Both groups tolerated the interventions well, with no notable adverse events related to the lidocaine infusion during or after surgery.

### 3.2. Outcomes

Following surgical intervention, blood levels of various cytokines and inflammatory markers were measured, including IL-6, IL-10, IL-17, TNF-α, IFN-γ, NE, CitH3, and MPO. The pre- and postoperative levels of the aforementioned markers are compared in Table 2. Additionally, BCR was evaluated.

There were significant differences in postoperative cytokine levels in MPO (*p* = 0.025) and IL-10 (*p* = 0.039), indicating a potential influence of lidocaine on these markers. However, no significant differences were observed in other cytokines, suggesting a selective effect of lidocaine on certain inflammatory markers.

In addition to the direct measurements of cytokine levels, we analyzed changes therein before and after surgery to assess the influence of lidocaine administration on the inflammatory response (Table 3). For each marker, the postoperative level was subtracted from the preoperative level to determine the change (delta, Δ) in the lidocaine and placebo groups.

Significant differences were observed in the changes in IL-10 and NE levels between the two groups, with the lidocaine group showing a greater increase in the IL-10 level and a smaller decrease in the NE level postoperatively. These results suggest potential anti-inflammatory and modulatory effects of lidocaine on certain cytokines and neuroendocrine factors. However, no significant group differences were observed in the changes in other markers, indicating a selective influence of lidocaine on inflammatory responses.

Additionally, univariable regression analysis is summarized in Supplementary Table S1. Among the cytokines analyzed, IL-10 (*p* = 0.017) and NE (*p* = 0.022) were significantly associated with changes, suggesting a potential modulatory effect of lidocaine on these markers.

## 4. Discussion

Recent research has increasingly focused on the role of cytokines in the tumor microenvironment of prostate cancer, highlighting their potential in modulating tumor growth, progression, and treatment response. Previous studies have explored the effects of cytokines such as IL-10 and molecules such as NE on prostate cancer. A meta-analysis demonstrated that IL10, a cytokine with anti-inflammatory properties, regulates immune responses within the tumor microenvironment, potentially affecting tumor progression and response to immunotherapies [11]. Similarly, NE, a serine protease with broad specificity that facilitates nonspecific bacterial clearance via the destruction of virulence factors on the cell membrane, influences tumor growth and metastasis via its effects on the tumor microenvironment, suggesting a link between stress, the immune response, and cancer progression [12]. 

MPO, an enzyme primarily produced by activated neutrophils, plays a crucial role in the innate immune system, particularly in the formation of reactive oxygen species, which are potent antimicrobials. However, recent studies have highlighted a potentially paradoxical role of MPO in the progression of several cancers, including prostate cancer [6]. Its involvement in prostate cancer is multifaceted, influencing inflammation, oxidative stress, and the tumor microenvironment. Elevated levels of MPO are associated with a higher tumor grade and stage in prostate cancer, suggesting its role as a prognostic biomarker [13]. Another study suggests that MPO plays a critical role in the inflammatory microenvironment of the prostate by promoting oxidative stress and cytokine release in prostate epithelial cells. Our findings show that perioperative lidocaine modulates MPO levels, potentially affecting inflammatory pathways. While MPO’s exact role in prostate cancer progression remains unclear, it is likely linked to its pro-oxidative and inflammatory effects rather than direct genotoxicity [14]. Therefore, its level can potentially guide therapeutic decision-making and risk stratification in clinical settings.

In prostate cancer, the tumor microenvironment is characterized by interactions with immune cells, such as neutrophils, playing a crucial role in disease development and progression. NETosis can contribute to cancer progression through several mechanisms. First, NETs enhance cell proliferation and tumor growth by inhibiting the growth factors and cytokines that promote a pro-tumorigenic environment. Second, components of NETs such as NE and MPO induce DNA damage and promote mutations in prostate cells, potentially leading to cancerous changes. Moreover, the physical structure of NETs can facilitate the formation of a scaffold that supports tumor cell adhesion and invasion, promoting metastasis. Additionally, the interaction between NETs and platelets can enhance thrombosis—a common complication in prostate cancer—which supports tumor spread and protects circulating tumor cells [6].

This study had several limitations. First, we enrolled only 60 participants from a single center. Second, only individuals from South Korea were enrolled. Future studies should enroll a larger sample from ethnically diverse populations. Third, this study focused on localized prostate cancer. Further research is needed to explore the effects on metastatic prostate cancers. Finally, we only enrolled patients who had undergone robot-assisted surgery, although the results may also be applicable to open surgery or laparoscopic radical prostatectomy.

While the study did not find a significant impact of lidocaine on BCR, previous research has reported that perioperative intravenous lidocaine administration significantly improved overall survival in bladder cancer via inflammatory responses [5]. Exploring the physiological mechanisms behind these differing results between the two studies could capture the interest of future scholars.

To the best of our knowledge, this is the first study to investigate the effects of perioperative lidocaine administration on serum inflammatory cytokines in patients undergoing radical prostatectomy for prostate cancer. Furthermore, we followed patients for an adequate duration of 2 years to determine BCR. Understanding the intricate roles of cytokines and neuroendocrine factors in prostate cancer could be important to identifying novel prognostic markers and therapeutic targets, thus facilitating personalized and effective treatment strategies for patients with localized prostate cancer. 

## 5. Conclusions

Until now, lidocaine has primarily been used in the field of urology as a local anesthetic for the rectum or perineum during prostate biopsies. Instead, this study demonstrates that perioperative lidocaine administration selectively modulates inflammatory and neuroendocrine responses in patients undergoing robot-assisted radical prostatectomy, particularly affecting MPO, IL-10, and NE levels without significantly altering other cytokines or biochemical recurrence rates. At the tested concentration, systemic lidocaine administration did not significantly affect oncological control, as evidenced by the similar rates of positive surgical margins, BCR, and time to BCR between the lidocaine and placebo groups. These findings suggest that while lidocaine may modulate certain inflammatory responses, it does not appear to influence cancer recurrence outcomes at the tested dosages. Our findings suggest the potential benefits of lidocaine in ERAS protocols and emphasize the need for further research to investigate its long-term impacts on cancer outcomes and recovery. This could contribute to optimized perioperative care and improved management strategies in oncologic surgeries.

## Figures and Tables

**Table 1:** Comparison of patient characteristics between the lidocaine and placebo groups.

	Lidocaine Group n = 30	Placebo Group n = 28	*p*-Value
Age (years)	66.43 ± 3.71	66.28 ± 5.93	0.709
BMI (kg/m^2^)	23.21 ± 1.29	23.73 ± 4.13	0.911
Diabetes mellitus	4	3	0.948
Hypertension	8	9	0.937
Hyperlipidemia	6	5	0.976
Cardiovascular disease	1	1	0.987
Cerebrovascular disease	1	1	0.987
Initial PSA level (ng/mL)	6.9 ± 3.3	6.7 ± 4.5	0.836
Pathological TNM stage (n)			
T2	30	28	0.773
Nx	20	21	0.828
N0	10	7	0.556
M0	30	28	0.773
Positive surgical margins (n)	2	1	0.804
BCR (n)	4	3	0.257
Time to BCR (months)	14.5 ± 4.5	24 ± 1.0	0.754

BCR = biochemical recurrence.

**Table 2:** Comparison of inflammatory cytokine levels before and after lidocaine administration during robotic radical prostatectomy in the lidocaine and placebo groups.

	Lidocaine Group	Placebo Group	*p*-Value
	n = 30	n = 28	
IL-6_Pre	6.4 ± 16.3	3.1 ± 8.4	0.323
IL-6_Post	44.6 ± 26.0	36.9 ± 29.9	0.293
IL-10_Pre	4.4 ± 7.0	5.2 ± 9.2	0.734
IL-10_Post	10.8 ± 13.2	5.1 ± 6.8	0.039
IL-17_Pre	1.7 ± 1.3	1.3 ± 1.0	0.130
IL-17_Post	1.8 ± 1.3	1.5 ± 1.8	0.385
TNF-α_Pre	8.6 ± 3.9	8.3 ± 3.8	0.775
TNF-α_Post	9.6 ± 4.3	8.2 ± 3.6	0.162
IFN-γ_Pre	4.4 ± 8.5	2.5 ± 2.8	0.242
IFN-γ_Post	2.3 ± 3.1	1.8 ± 1.2	0.405
NE_pre	25.0 ± 24.3	22.6 ± 19.5	0.674
NE_post	27.0 ± 28.0	21.3 ± 18.6	0.368
CitH3_pre	146.4 ± 23.1	143.7 ± 17.1	0.615
CitH3_post	144.4 ± 28.9	140.8 ± 15.9	0.558
MPO_pre	3.2 ± 2.0	3.8 ± 1.5	0.161
MPO_post	2.6 ± 1.6	3.5 ± 1.4	0.025

IL = interleukin; TNF-α = tumor necrosis factor-α; IFN-γ = interferon-γ; NE = neutrophil elastase; CitH3 = citrullinated histone3; MPO = myeloperoxidase.

**Table 3:** Comparison of changes in inflammatory cytokine levels before and after lidocaine administration during robotic radical prostatectomy in the lidocaine and placebo groups.

	Lidocaine Group	Placebo Group	*p*-Value
	n = 30	n = 28	
ΔIL-6	−38.2 ± 18.5	−32.7 ± 30.8	0.397
ΔIL-10	−5.7 ± 5.9	−0.7 ± 0.6	0.001
ΔIL-17	−0.1 ± 0.02	−0.2 ± 0.04	0.821
ΔTNF-α	−1.0 ± 0.2	0.4 ± 0.3	0.126
ΔIFN-γ	2.1 ± 1.3	0.7 ± 0.4	0.274
ΔNE	−1.1 ± 0.4	2.0 ± 0.8	0.022
ΔCitH3	6.8 ± 5.2	7.5 ± 6.3	0.932
ΔMPO	0.7 ± 2.2	0.4 ± 1.2	0.602

Δ = pretreatment cytokine level—posttreatment cytokine level; IL = interleukin; TNF-α = tumor necrosis factor; IFN-γ = interferon-γ; NE = neutrophil elastase; CitH3 = citrullinated histone3; MPO = myeloperoxidase.

## Data Availability

The raw data supporting the conclusions of this article will be made available by the authors on request.

## Supplementary Materials

The following supporting information can be downloaded at https://www.mdpi.com/article/10.3390/medicina60091452/s1. Table S1. Univariable regression analyses to assess changes in cytokines and BCR with the lidocaine injection during the robot radical prostatectomy.
