# Supplementary material for: Impact of Perioperative Lidocaine on Neutrophil Extracellular Trapping and Serum Cytokines in Robot-Assisted Radical Prostatectomy: Randomized Controlled Study
Source: Medicina (Kaunas). 2024 Sep 5;60(9):1452. doi: 10.3390/medicina60091452 (PMC11434533; doi:10.3390/medicina60091452)
Supplement: Supplementary file 1 [file medicina-60-01452-s001.zip › medicina-3173116-supplementary.pdf]

Supplementary Table S1. Univariable regression analyses to assess changes in cytokines and BCR with the lidocaine injection during the robot radical prostatectomy

Variables	Coefficient (95% CI)	*p*-value
IL6	-0.75 (-1.67, 0.17)	0.397
IL10	5.49 (0.99, 9.98)	0.017
IL17	-0.75 (-1.67, 0.17)	0.113
TNF	1.45 (-0.42, 3.31)	0.125
IFNr	-1.37 (-3.81, 1.07)	0.274
NE	3.05 (0.46, 5.65)	0.022
CitH3	0.72 (-15.97, 17.40)	0.932
MPO	0.24 (-0.69, 1.17)	0.602
BCR	0.29 (-1.30, 1.88)	0.727

interleukin-6 (IL6), interleukin-10 (IL10), interleukin-17 (IL17), tumor necrosis factor-α (TNF), interferon-γ (IFNr), and norepinephrine (NE), citrullinated histone (CitH3), myeloperoxidase (MPO), biochemical recurrence (BCR)
